# Reduced serum iron levels are associated with metabolic dysfunction and sex-specific characteristics

**DOI:** 10.1007/s11739-025-04169-x

**Published:** 2025-11-06

**Authors:** Antonio Francesco Maria Giuliano, Carlo De Matteis, Salvatore Cantatore, Ersilia Di Buduo, Fabio Novielli, Elsa Berardi, Gianfranco Antonica, Antonio Moschetta, Lucilla Crudele

**Affiliations:** 1 Department of Interdisciplinary Medicine, University of Bari “Aldo Moro”, 70124 Bari, Italy; 2 Department of Precision and Regenerative Medicine and Ionian Area (DiMePre-J), Clinica Medica “A. Murri”, University of Bari “Aldo Moro” Medical School, Bari, Italy; 3 INBB National Institute for Biostructure and Biosystems, Viale Delle Medaglie d’Oro 305, 00136 Rome, Italy

**Keywords:** Metabolic syndrome, Cardiovascular risk, Liver steatosis, Obesity, Ferroptosis, Gender medicine

## Abstract

Metabolic syndrome (MetS) is a clinical condition defined by abdominal obesity, insulin resistance, hypertension, and dyslipidaemia, associated with increased cardiovascular risk and type 2 diabetes (T2D). Emerging evidence suggests a role for iron metabolism and ferroptosis in the pathophysiology of MetS and its related complications. This study aimed to explore the association between serum iron levels and clinical, anthropometric, and biochemical parameters in a population with cardiometabolic risk factors. Weongoing treatment for such analysed data from 893 patients attending the Metabolic Disease Unit of the Interdisciplinary Medicine Department at the University of Bari “Aldo Moro”. Patients with MetS, elevated BMI, hypertension, and T2D exhibited significantly lower serum iron levels compared to healthy controls. Serum iron showed a strong inverse correlation with age (*r* = − 0.09, *p* = 0.0061), fasting plasma glucose (*r* = − 0.10, *p* =  = .002), and HbA1c (*r* = − 0.18, *p* < 0.0001) in the overall population, while no correlations were found with Framingham Risk Score, triglycerides, waist circumference, and BMI. Conversely, when stratifying by sex, we observed that serum iron was inversely correlated with BMI (*r* = − 0.12, *p* = 0.008) and waist circumference (*r* = − 0.12, *p* = 0.008) in females only. Metabolic dysfunction is associated with reduced serum iron levels, with sex-specific patterns observed in relation to adiposity markers. Elucidating the interplay between iron metabolism, sex hormones, and adipose tissue biology may uncover new targets for personalized treatment strategies for metabolic diseases. Further research is warranted to clarify how modulation of iron homeostasis affects adipose tissue function, particularly in women with obesity and related metabolic disorders.

**Graphical abstract:**

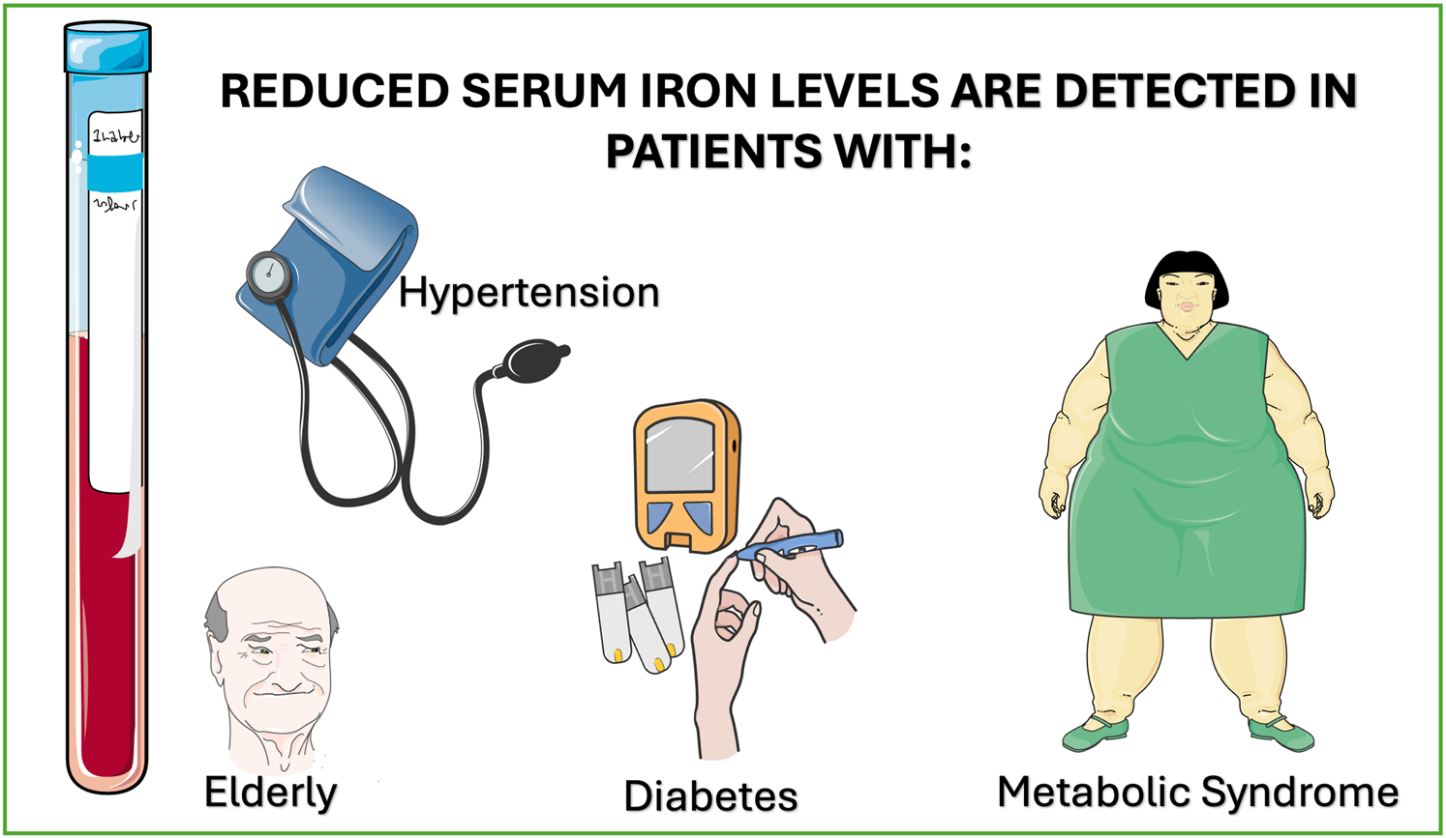

**Supplementary Information:**

The online version contains supplementary material available at 10.1007/s11739-025-04169-x.

## Introduction

Iron plays a pivotal role in numerous physiological processes, including oxygen transport, mitochondrial function, and cellular metabolism. Ferroptosis**,** a form of programmed cell death driven by iron overload, glutathione-dependent antioxidant system failure, and lipid peroxidation, has been proposed as a cellular mechanism linking iron dysregulation to adverse cardiovascular outcomes [1]. Several studies have highlighted the contribution of tissue iron overload to the generation of reactive oxygen species (ROS), lipid peroxidation, and lipid accumulation in multiple organs [2]. Consequently, ferroptosis has been implicated in pancreatic β-cell dysfunction, contributing to the development of type 2 diabetes (T2D) [3], as well as in the pathogenesis of metabolic dysfunction-associated steatotic liver disease (MASLD) [4]. These metabolic disorders are often co-present within the clinical entity known as metabolic syndrome (MetS), which arises from a complex interplay of abdominal obesity, insulin resistance, hypertension, and dyslipidaemia [5]. This constellation of metabolic abnormalities significantly increases the risk for T2D and various cardiovascular diseases (CVDs). Recent epidemiological data indicate that MetS affects approximately 25–33% of the global adult population [6, 7], posing a substantial burden on healthcare systems, patient quality of life, and socioeconomic structures [8]. A variety of environmental, genetic, societal, and dietary factors contribute to the onset, progression, and management of MetS. Current research efforts are increasingly focused on elucidating the metabolic pathways and identifying biomarkers involved in its pathogenesis [9]. In this context, circulating iron parameters—including ferritin, transferrin, and hepcidin—have been investigated for their association with MetS and CVD. Although serum ferritin levels have been found increased in patients with MASLD and liver steatosis, chronic inflammation, obesity, and dyslipidaemia [10], little is known on iron serum levels in subjects with cardiometabolic risk conditions.

For instance, iron deficiency is frequently observed in patients with heart failure and is associated with worse clinical outcomes, including higher rates of hospitalization and mortality [11]. Randomized trials have demonstrated that correction of iron deficiency can improve symptoms and reduce adverse cardiovascular events in these patients [12, 13]. Conversely, iron overload may contribute to vascular injury and atherogenesis by promoting endothelial dysfunction and plaque instability through enhanced ROS production [14]. Moreover, genetic studies suggest that polymorphisms affecting systemic iron regulation are associated with coronary artery disease risk, underscoring the complex and bidirectional role of iron homeostasis in cardiovascular health [15].

Thus, in this observational study, we primarily aimed to investigate the association between serum iron levels and the prevalence of MetS in a large cohort of patients with cardiometabolic risk factors. Furthermore, we also explored the correlation between iron levels and the clinical entities contributing to cardiometabolic risk, with a focus on potential sex-specific differences.

## Materials and methods

### Study participants

Written informed consent for the use of clinical data was obtained from all participants in the study. The study was approved by the Ethics Committee (n.311, MSC/PBMC/2015) of the Azienda Ospedaliero-Universitaria Policlinico di Bari (Bari, Italy) in accordance with the requirements of the Declaration of Helsinki. The initial study population enrolled patients whose clinical and biochemical parameters were collected in the electronic health register of the Metabolic Disease Department in Interdisciplinary Medicine—Internal Division “Cesare Frugoni” of the “Aldo Moro” University of Bari, Policlinico (Bari, Italy) from 2017 to June 2024. A total of 2,767 observations were initially considered; we then excluded 1,222 observations because data about iron, serum ferritin, and blood count values were not available. We further excluded 647 evaluations consisting of re-evaluations for the same patients, patients with genetic hemochromatosis, and subjects having iron-supplementation therapy. Additionally, five subjects were removed from the final cohort, since their iron levels were considered outliers according to Grubbs’ outlier test. Finally, we performed statistical analysis on a population of 893 patients with a quite similar distribution of two sexes (48% males, 52% females) (Supplementary Fig. 1).

### Clinical and biochemical assessment

Physical examination, anthropometric measures, biochemical assessment, and abdomen ultrasound were performed. Morning blood samples were obtained after 12 h of fasting from the antecubital veins of patients. After blood clotting and centrifugation, serum was processed for analysis of biochemical markers of lipid and glucose metabolism. All biochemical measurements were centralized and performed in the ISO 9001 certified laboratories of the University Hospital of Bari. Specifically, blood count, haemoglobin and iron blood concentration, and serum ferritin were measured. Measurements of total and HDL cholesterol, fasting plasma glucose (FPG), and triglycerides were obtained through enzymatic colorimetric assay (Siemens, Erlangen, Germany). Glycosylated haemoglobin (HbA1c) was assessed in human whole blood using ion-exchange high-performance liquid chromatography (HPLC) on the Bio-Rad Variant II Haemoglobin A1c Program (BIO-RAD Laboratories Srl, Milan, Italy). Iron and ferritin measurements were carried out on fully automated clinical analysers and serum ferritin by immunoassays, whereas serum iron was measured using a colorimetric reaction. After an overnight fasting, patients underwent an abdominal ultrasound scanning with a 3.5–5 MHz convex probe (Esaote My Lab 70 Gold ultrasound system). B-mode ultrasound was used for assessment of fatty liver. Mild steatosis was defined by diffusely increased hepatic echogenicity, but periportal and diaphragmatic echogenicity was still appreciable. Moderate steatosis was represented by a diffusely increased hepatic echogenicity obscuring periportal echogenicity, but diaphragmatic echogenicity was still appreciable. Severe steatosis was diagnosed when hepatic echogenicity was diffusely increased, obscuring periportal as well as diaphragmatic echogenicity. MetS was diagnosed according to the NCEP ATP III definition; visceral obesity was defined for waist circumference (WC) values equal to or above 88 cm in women and 102 cm in men. More isolated metabolic criteria were considered: impaired glycaemic and lipid control and arterial hypertension. Average systolic and diastolic blood pressures (BPs) were recorded for each patient in three different measurements using a manual sphygmomanometer. Hypertension was diagnosed for systolic BP ≥ 130 mmHg, diastolic BP ≥ 85 mmHg, and/or treatment with antihypertensive agents. Impaired glycemic control was diagnosed for FPG>110 mg/dL, impaired lipid control was diagnosed for HDL cholesterol <40 mg/dL in males and <50 mg/dL in females, and for triglycerides >150 mg/dL, or ongoung treatment for such impairements. For T2D, the criteria were HbA1c ≥ 48 mmol/mol, FPG ≥ 126 mg/dL, or glycaemia > 200 mg/dl at 2 h during oral glucose test tolerance and/or treatment for diabetes. BMI (body mass index) was computed as weight (kg) divided by the height (m) squared, and BMI values (kg/m^2^) 25.0–29.9 and over 30.0 were considered as overweight and obesity conditions, respectively. The Framingham Risk Score was used to calculate the 10-year risk for cardiovascular events. MASLD diagnosis was based on the presence of liver steatosis identified by ultrasound and at least one of the five criteria for MetS, also considering BMI ≥ 25 kg/sqm to assess overweight or obesity as an alternative to increased WC. Ultrasound assessment of internal carotid arteries was used to detect atherosclerotic disease, permitting the evaluation of both the macroscopic appearance of plaques and flow characteristics in the carotid artery.

### Data analysis

Grubbs’ outlier test was used to detect outliers. Descriptive statistical analyses of the study sample were performed, and results were expressed as mean ± standard deviation (SD) for continuous variables and percentages for categorical ones. Data distribution was assessed using the Kolmogorov–Smirnov test. As the data were found to be normally distributed, parametric tests were used for the subsequent statistical analyses. Comparisons of clinical variables between two groups were conducted with Student’s *T* test, while ANOVA was performed to assess differences among three or more groups. All reported *p*-values (*p*) were based on two-sided tests and compared to a significance level of 5%. The significance level for all statistical tests was set a priori at α = 0.05. The magnitude of the differences was assessed by calculating Cohen's d for the Student's *T* test and partial eta-squared (η_p^2) for ANOVA. Data were represented by using box plots showing the median (second quartile), first, and third quartiles, and whiskers representing minimum and maximum values. Correlations between iron levels and bio-humoral and clinical parameters were analysed and estimated using Pearson’s correlation coefficient (*r*). Correlations were shown in heatmaps with *R* values and respective *p*-values. Receiver operating characteristic (ROC) curve was performed to identify the association between lower iron serum levels and MetS. The area under the curve (AUC) was plotted to distinguish between clinical groups and Youden’s Index, or equivalently, the highest sensitivity + specificity was used to determine the optimal cutoff of each variable for the prediction of MetS. Analysis of covariance (ANCOVA) was used to compare group means while adjusting for the influence of continuous covariates. Potentially confounding variables in the assessment of the causal effect were accounted for in a multivariable logistic regression and were selected a priori based on their established roles as risk factors for metabolic disease and their potential influence on iron metabolism, as documented in existing literature. All analyses were performed using the NCSS 2023 Statistical Software (2023, NCSS, LLC, Kaysville, Utah, USA) and GraphPad Prism, version 10 (GraphPad Software; San Diego, CA, USA).

## Results

### Study population characterization

The mean age of the subjects was 58.7 ± 14.6 years. Overall, subjects displayed increased BMI (64%) and abdominal obesity (54%) according to diagnostic criteria for MetS that was diagnosed in 449 subjects (54%). Dysmetabolic conditions such as atherosclerosis, arterial hypertension, liver steatosis, and T2D were also largely represented in our population study. According to Framingham Risk Score (mean 18.8 ± 16.8), our population had an intermediate risk of developing coronary heart disease within 10 years. Mean iron (83.7 ± 29.4 mcg/dL) and serum ferritin (98.9 ± 90.9 ng/mL) levels were both normal. While mean FPG and HbA1c depicted a condition of pre-diabetes, lipid panel did not show noteworthy alteration. The details are shown in Table 1.

**Table 1:** Study population characterization

Sample number (m;f)	893 (427;466)
Age (years)	58.7 ± 14.6 (39)
BMI (kg/m^2^)	27.2 ± 5.3 (14.8)
Waist circumference (cm)	97.2 ± 14.5 (43)
Iron (mcg/dL)	83.7 ± 29.4 (59)
Serum ferritin (ng/mL)	98.9 ± 90.9 (285)
Erythrocytes (× 10^6^/mL)	4.8 ± 0.5 (2.4)
Haemoglobin (g/dL)	13.9 ± 1.4 (6.5)
Platelet count (× 10^3^/μL)	253.3 ± 65.1 (128)
Glucose (mg/dL)	99.4 ± 25.9 (101)
HbA1c (mmol/mol)	40.2 ± 10.1 (14.3)
Total cholesterol (mg/dL)	180.4 ± 41.6 (140)
HDL cholesterol (mg/dL)	54.3 ± 14.5 (72)
LDL cholesterol (mg/dL)	106.4 ± 36.1 (149)
Triglycerides (mg/dL)	113.7 ± 60.8 (186)
CRP	5.2 ± 12.5 (200)
ERS	19.8 ± 16.7 (135)
Framingham risk score	18.9 ± (33.5)
Smokers (*n*;%)	286 (32)
Abdominal obesity (*n*;%)	475 (54)
Overweight (BMI = 25–29.9) (*n*;%)	331 (38)
General obesity (BMI > 29.9) (*n*;%)	224 (26)
Metabolic syndrome (*n*;%)	449 (50)
Atherosclerosis (*n*;%)	365 (58)
Arterial hypertension (*n*;%)	569 (66)
Liver steatosis (*n*;%)	510 (61)
Type 2 diabetes (*n*;%)	346 (39)

Data are presented as mean ± SD with range (in round brackets) for continuous variables, and in number and percentages for categorical ones

*m* males, *f* females, *CRP* C-reactive protein, *ESR* erythrocyte sedimentation rate

**Fig. 1:**
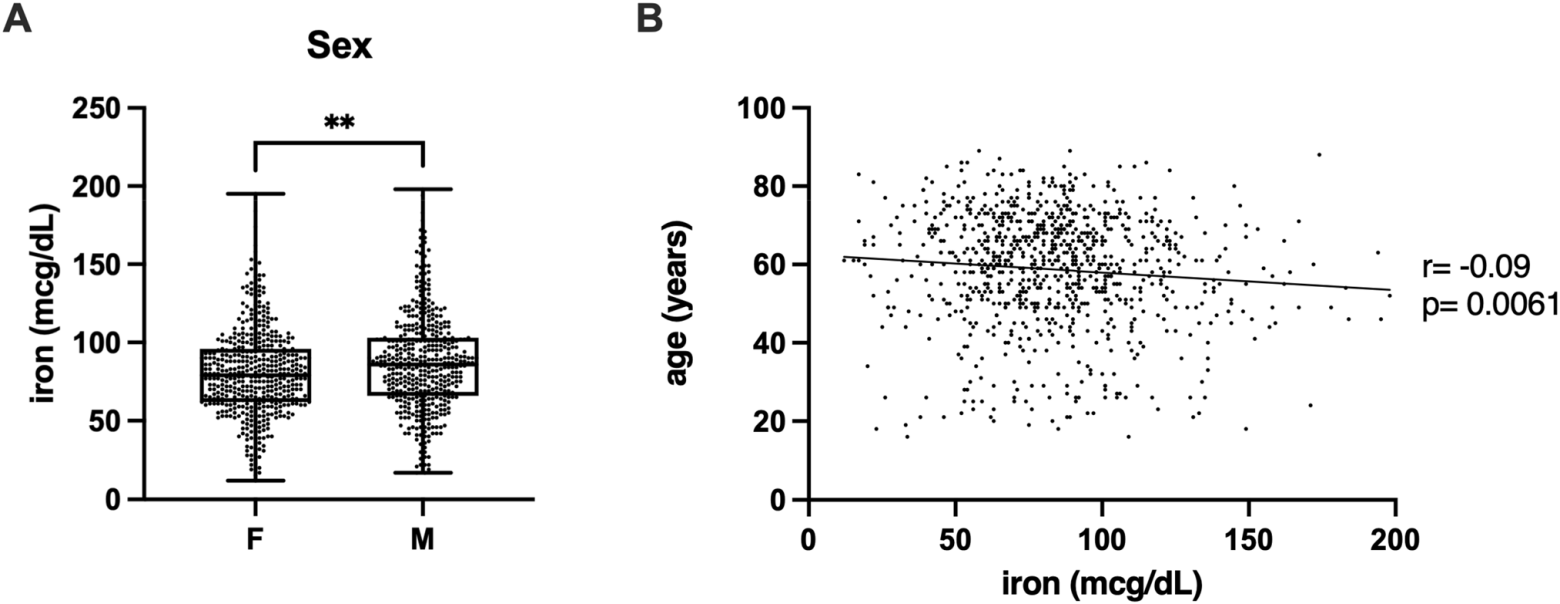
Iron levels according to sex and age. (**A**) Student’ T test was performed to assess differences between the two groups. *p* < 0.05 was considered significant. The box plots show the median (second quartile), first, and third quartile, and whiskers represent minimum and maximum values. **p < 0.01. (**B**) Pearson’s correlations (*r*) and the corresponding *p*-values (*p*) are reported

We compared iron concentration in two sexes (Fig. 1a), finding that iron levels were significantly higher (*p* = 0.0012, Cohen’s d = 0.74) in males (87.1 ± 30.6) than in females (80.6 ± 27.9). We also studied correlation between age and iron values (Fig. 1b), finding a significant (*p* = 0.0061) inverse correlation (*r* = − 0.09). Moreover, since women generally experience increased iron levels due to cessation of menstruation, we divided our female population into two groups according to age under and above 55 years, finding no significant difference between younger and older women (82.1 ± 31.3 vs 79.7 ± 25.6, *p* = 0.3960).

### Iron level correlation with cardiovascular risk

**Fig. 2:**
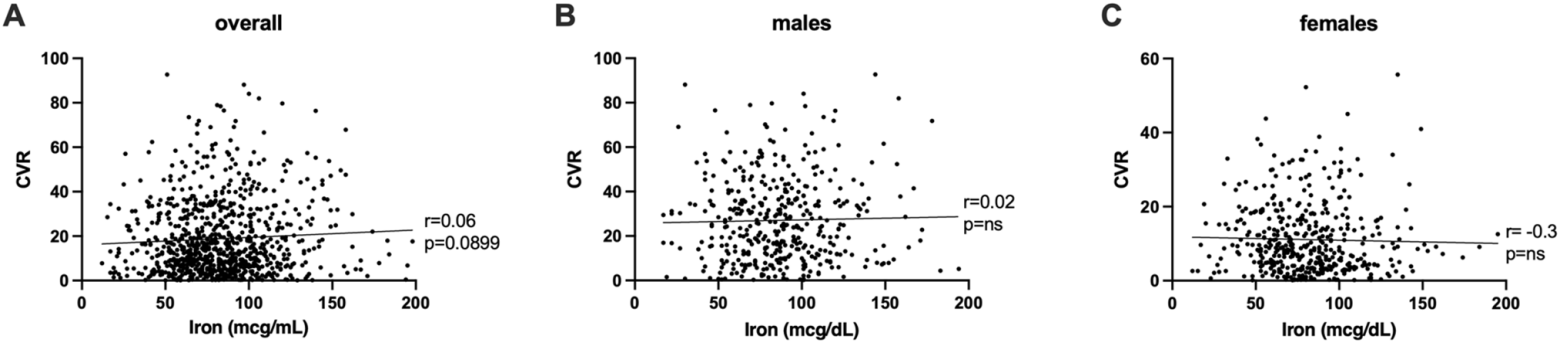
Iron level correlations with cardiovascular risk (CVR) assessed by the Framingham Risk Score. Pearson’s correlations (*r*) and the corresponding p-values (*p*) are reported

Aiming to investigate whether iron level could be involved in determining an increased cardiovascular risk (CVR), we firstly studied its correlations with Framingham Risk Score. We performed Pearson correlation in the overall population (Fig. 2a), finding no significant correlation between iron levels and CVR (*p* = 0.0899). Considering known CVR differences between males and females, we then performed different correlation studies in the two sexes (Fig. 2b–c). Such studies confirmed that no significant correlation between Framingham Risk Score and iron levels could be identified in our population.

### Iron level comparison in patients with and without cardiometabolic risk factors

**Fig. 3:**
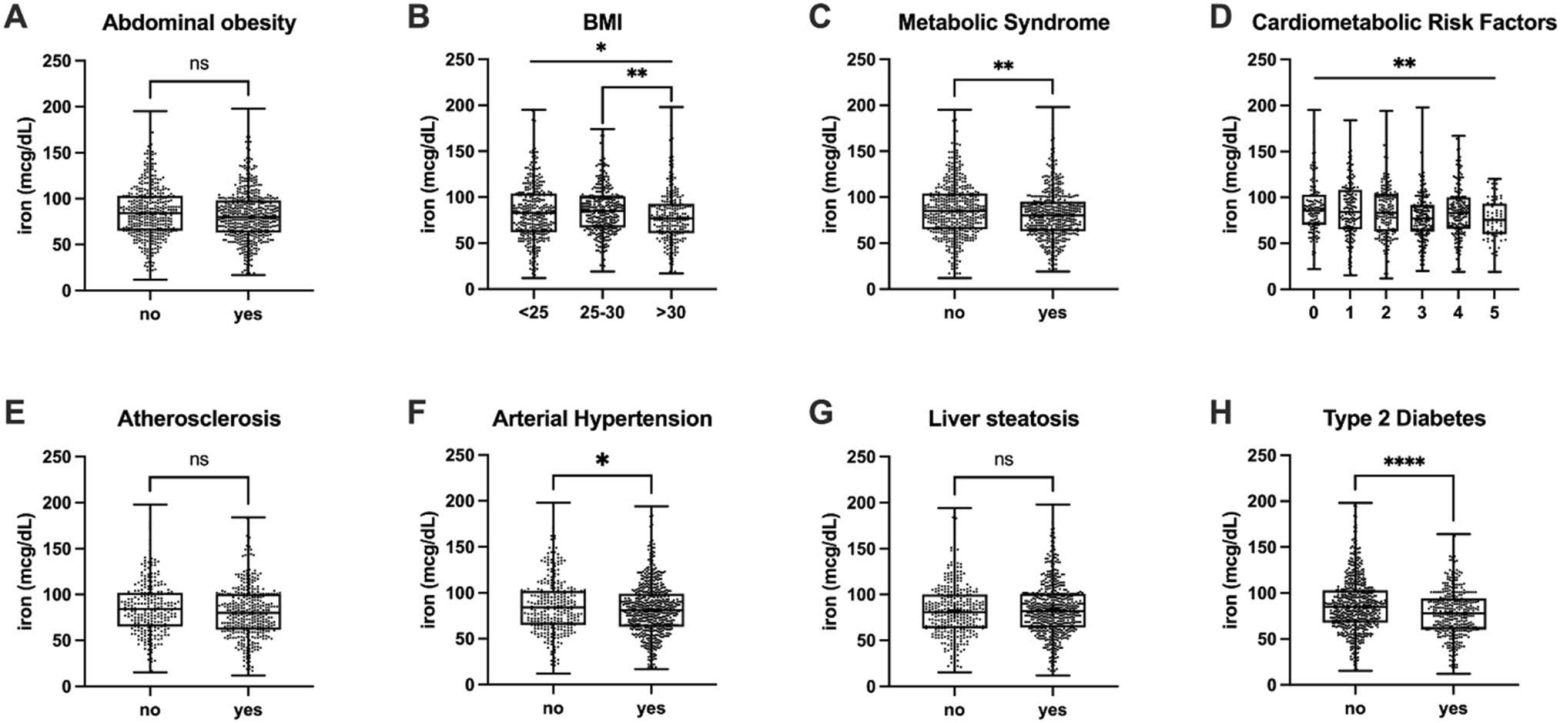
Comparison of iron level in patients with and without dysmetabolic conditions. Student’ *T* test was performed to assess differences between two groups. ANOVA was performed to assess differences among three or more groups. *p* < 0.05 was considered significant. The box plots show the median (second quartile), first, and third quartile, and whiskers represent minimum and maximum values. *p < 0.05; **p < 0.01; ****p < 0.0001; ns = not significant

To investigate whether iron level differs in subjects with specifical clinical and dysmetabolic conditions, we firstly compared iron levels in patients with and without cardiometabolic risk factors. We did not find any difference when comparing patients with and without increased WC, although a trend could be identified (82.0 ± 28.1 vs 85.3 ± 29.9, *p* = 0.1761, Cohen’s d = 0.23) (Fig. 3a), while a significant decrease was found considering different classes of BMI (ANOVA *p* = 0.0145, η^2^p = 0.07). Specifically, iron levels were significantly lower in subjects with obesity (78.8 ± 28.6) compared to overweight subjects (86.4 ± 27.0) (*p* = 0.0017, Cohen’s d = 0.71) (Fig. 3b). We then investigated iron level differences in patients with and without Mets (Fig. 3c), finding significant decreased levels in metabolic patients (86.8 ± 31.2 vs. 80.6 ± 27.0, *p* = 0.0017, Cohen’s d = 0.63).

Since we found significant difference when comparing iron levels in patients according to the absence or presence of different number of criteria to diagnose MetS (Fig. 3d, p = 0.009, η^2^p = 0.09), we investigated if iron levels could be associated with specific MetS clinical features. Thus, we compared subjects with and without atherosclerosis (Fig. 3e), finding no significant difference in (p = 0.0539, Cohen’s d = 0.38) iron levels in patients with atherosclerotic plaque (81.4 ± 29.2) compared to subjects without any vessel alteration (86.1 ± 30.1), while such difference was significant when dividing our study population according to arterial hypertension diagnosis (86.4 ± 30.5 vs. 82.3 ± 28.8, *p* = 0.0484, Cohen’s d = 0.42) (Fig. 3f). Also, differences between subjects with and without liver steatosis (Fig. 3g) were not significant (83.8 ± 31.7 vs 84.9 ± 33.2, *p* = 0.5837, Cohen’s d = 0.11). Conversely, the strongest significant difference (*p* < 0.0001, Cohen’s d = 0.89) was found when comparing patients with and without diabetes (Fig. 2h). Indeed, diabetic patients displayed significantly lower iron levels (78.5 ± 26.7) compared to subjects without diabetes (87.1 ± 30.3).

To assess for multicollinearity prior to the multivariate analysis, we tested the correlation between serum iron and serum ferritin, finding a non-significant association (*r* = 0.05, *p* = 0.1181). Therefore, both variables were retained in the following analyses. Thus, we investigated the role of confounding factors in determining the association of low iron levels with MetS. Through ROC curve (sensibility 75%, specificity 42%, *p* = 0.0013), the iron level cutoff of 94 mcg/dL to diagnose MetS was calculated. Thus, we performed a logistic regression to analyse the risk for MetS in patients with lower iron levels.

**Fig. 4:**
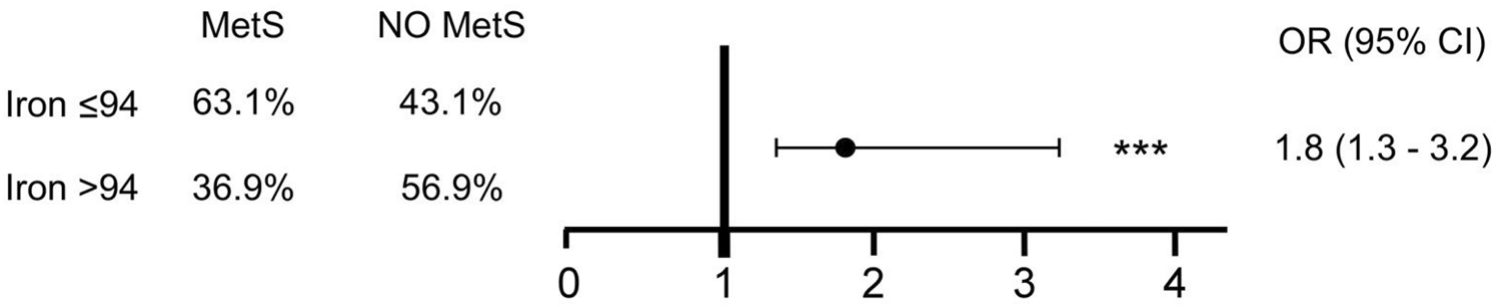
Multivariate analyses for low iron levels and metabolic syndrome (MetS). Logistic regression was performed to investigate the association after adjusting for age, sex, smoking, physical activity, inflammatory markers ESR and CRP, and serum ferritin. ***p < 0.001

We observed that such relationship was still significant following the adjustment for covariates like age, sex, smoking, physical activity, serum ferritin levels, ESR, and CRP as markers of inflammation (Fig. 4). Compared to individuals with higher iron levels, those with lower values had significantly higher OR of being classified as having MetS (OR = 1.8; *p* < 0.001).

### Iron level correlations with bio-humoral parameters in the two sexes

**Fig. 5:**
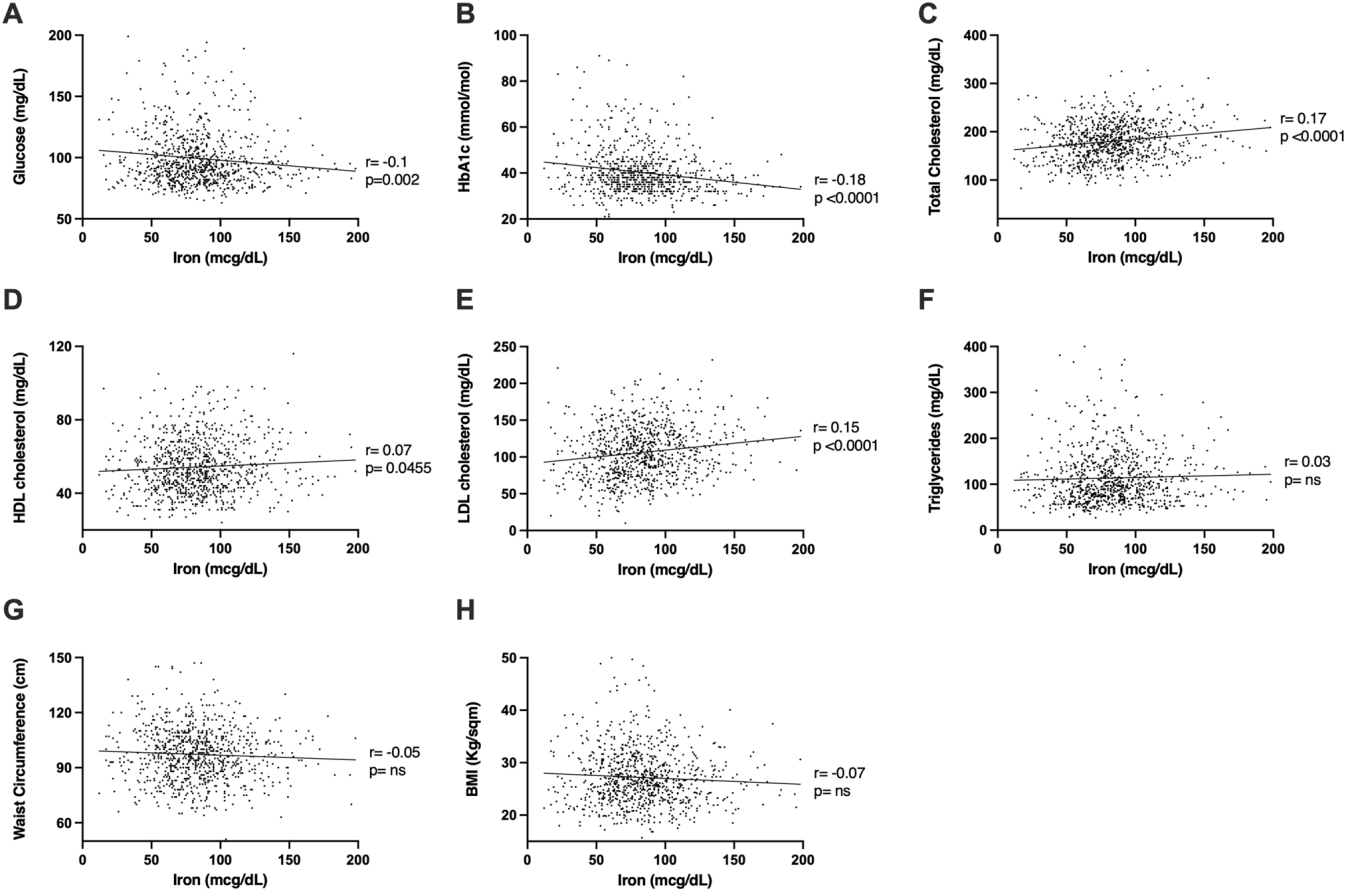
Iron level correlations with bio-humoral and clinical parameters in the study population. Pearson’s correlations (*r*) and corresponding *p*-values (*p*) are reported

To deepen the iron putative role in determining CVD and metabolic syndrome-associated diseases, we then performed a correlation study between iron levels and bio-humoral parameters considered as diagnostic criteria. We found that in the overall population (Fig. 5), iron levels were significantly and inversely correlated with FPG (*r* = − 0.1, *p* = 0.002) and HbA1c (*r* = − 0.18, *p* < 0.0001), suggesting an involvement of iron levels in glucose metabolism. We then considered lipidic profile, finding that iron levels were significantly correlated with total cholesterol (*r* = 0.17, *p* < 0.0001), HDL-cholesterol (*r* = 0.07, *p* = 0.0455), and LDL-cholesterol (*r* = 0.15, *p* < 0.0001), while triglycerides did not display a statistically significant correlation (*p* = 0.3246). Then, we performed a correlation analysis also for anthropometric measures, finding that neither BMI (*p* = 0.0662) nor WC (*p* = 0.1243) was significantly correlated with iron values. After adjusting for serum ferritin, such correlations were confirmed, and WC was found to be inversely correlated with iron levels (Supplementary Table 1). We also performed serum ferritin correlation analysis with bio-humoral and clinical parameters, showing that it positively and significantly correlated with WC, BMI, triglycerides, and LDL cholesterol (Supplementary Fig. 2).

**Fig. 6:**
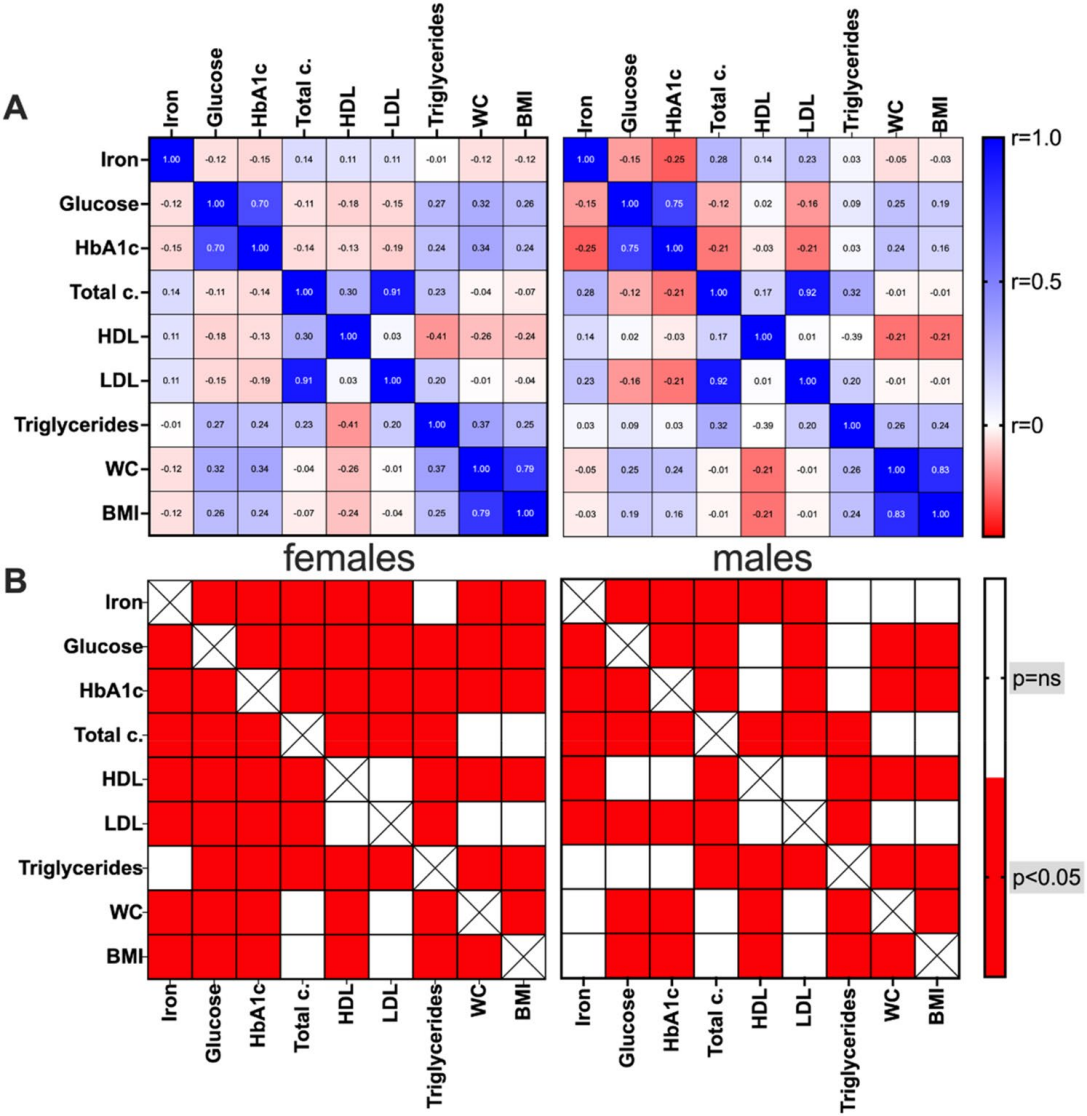
Correlations among bio-humoral and clinical parameters in females and males. Heatmap representation of R values (**A**) and the respective p-values (**B**) of Pearson’s correlations among bio-humoral and clinical parameters in females and males. Lateral bars show colours in the legend. The brightness of the colours indicates how strong the correlation is (**A**). *p* < 0.05 were considered significant and are indicated as red squares (**B**)

Finally, we analysed iron correlations separately in the two sexes (Fig. 6). While iron correlations with glycaemic and lipidic parameters were confirmed, some intriguing differences were discovered between males and females regarding anthropometric parameters. Specifically, differently from the overall population, in women an inverse significant correlation was present for both WC (*r* = − 0.12, *p* = 0.008) and BMI (*r* = − 0.12, *p* = 0.008).

## Discussion

Over the past decade, growing evidence has investigated the role of iron metabolism in modulating cardiovascular risk. In the present study, we specifically deepened the role of iron serum levels as marker of acquired metabolic disease. We observed no significant correlation between serum iron levels and the Framingham Risk Score, one of the most widely validated tools for predicting cardiovascular risk. However, a more detailed analysis revealed that abnormal iron levels were significantly associated with specific cardiometabolic risk factors. Lower serum iron levels were observed in patients with elevated BMI, MetS, arterial hypertension, and T2D. These findings suggest that iron metabolism may contribute to the pathophysiological mechanisms underlying the clinical components of MetS. This association is likely mediated through iron’s role in promoting inflammation and tissue injury via oxidative stress pathways [16].

In our study, we observed significant inverse correlations between serum iron levels and both fasting glycaemia and HbA1c. These findings suggest that iron deficiency may influence the pathophysiology of T2D, potentially exacerbating insulin resistance and impairing glycaemic control. Consistent with our results, in patients with iron deficiency anaemia, HbA1c decreased significantly after iron treatment [17], as well as a previous cross-sectional study involving 143 adult patients with diabetes reported an association between iron deficiency anaemia and altered glycaemic control [18].

Similarly, an interventional clinical trial demonstrated that iron supplementation significantly reduced fasting blood glucose and HbA1c levels, thereby improving insulin resistance in women with T2D [19]. This sex-specific effect was further supported by a retrospective study showing that 39.3% of individuals with T2D had iron deficiency anaemia, with higher prevalence among women and those with longer disease duration. Independent risk factors for anaemia included female sex, longer duration of diabetes, and elevated fasting plasma glucose levels [20].

Aligned with the sex-specific role of iron in diabetes, we found that obesity—assessed using both BMI and WC—was significantly and inversely correlated with serum iron levels in women, but not in men. Supporting our findings, a large multicentre population-based study conducted across four European countries reported a significantly higher prevalence of iron deficiency anaemia in women compared to men, primarily attributed to menstrual blood loss. Additional risk factors in both sexes included low dietary iron intake, gastrointestinal blood loss, and chronic disease [21].

Furthermore, our findings corroborate evidence from a cross-sectional study showing a significantly higher risk of iron deficiency in women with the highest body fat percentage (BF%) compared to those with the lowest BF% [22]. Similarly, lower serum iron levels have been reported in women with obesity compared to those with normal body weight [23].

If previous evidence suggests a relationship between low serum iron levels and obesity, the biological underpinnings of such sex-specific findings warrant further investigation. Adipose tissue is not merely a fat storage depot, but an active endocrine organ that plays a critical role in systemic energy homeostasis. Men and women exhibit distinct patterns of adipose tissue distribution and function, with women generally having greater subcutaneous fat and men presenting with increased visceral adiposity [24]. These differences are largely regulated by sex hormones, which also influence iron metabolism.

Indeed, fluctuations in oestrogen and testosterone levels modulate not only iron absorption, storage, and mobilization, but also adipocyte differentiation, lipid storage, and the inflammatory milieu within adipose tissue. These hormone-driven processes impact systemic metabolic pathways, including insulin sensitivity and lipid metabolism.

Importantly, iron availability within adipocytes influences mitochondrial activity and adipogenesis—processes essential for the healthy expansion and metabolic function of adipose tissue [25]. Dysregulation of iron metabolism can lead to oxidative stress and chronic inflammation, contributing to adipose tissue dysfunction [26]. Of note, individuals with obesity frequently display altered iron homeostasis, characterized by reduced serum iron levels despite normal or elevated total body iron stores. This paradox is partially explained by inflammation-induced upregulation of hepcidin, which restricts iron mobilization from stores [27]. In this context, the typically lower serum iron levels observed in women—including in our cohort—may differentially affect adipose tissue metabolism, potentially influencing adipose tissue expansion, insulin sensitivity, and inflammatory responses. Conversely, men’s relatively higher serum iron levels may predispose to increased oxidative stress, particularly in visceral fat, thereby contributing to a greater cardiometabolic risk.

Moreover, the chronic low-grade inflammation associated with increased visceral adiposity in men may alter the relationship between anthropometric measurements and serum iron levels. This sex-specific inflammatory environment could account for the absence of significant correlations between serum iron and anthropometric markers in men. It may also help explain why, in our stratified analysis, serum iron levels were significantly associated with BMI, but not with WC in the general population.

Furthermore, oestrogens are known to influence iron homeostasis by regulating the expression of hepcidin, the key hormone controlling systemic iron absorption and distribution [28]. Hepcidin acts by inhibiting intestinal iron absorption and the release of iron from macrophages, thereby contributing to functional iron deficiency [29, 30]. Within macrophages, iron is stored as ferritin and released as needed via the iron exporter ferroportin.

During inflammatory states, hepatic production of hepcidin is upregulated, leading to downregulation of ferroportin. This process impairs the transfer of dietary iron from enterocytes in the small intestine into the bloodstream and restricts the release of recycled iron from macrophages located in the spleen and liver, potentially resulting in iron deficiency [31]. Conversely, ferritin—an acute-phase reactant—increases during both acute and chronic inflammation, as well as oxidative stress. As also calculated in our population, elevated ferritin levels have been reported in T2D, CVD [32], MetS, and its clinical components [33] with higher concentrations being associated with increased odds of these conditions [34]; notably, the rise in ferritin levels and its associated disease risk appears more pronounced in females [35].

Nevertheless, to clarify this relationship, further molecular, genomic, and histopathological studies are required also in view of some contrasting evidence. For instance, a large cross-sectional study in a Chinese population [36] found that higher levels of serum iron were positively associated with the prevalence of MetS. However, some factors could explain this discrepancy. First, our population was composed of patients who already presented with a high prevalence of cardiometabolic risk factors. Second, ethnic variations in genetics, diet, and iron metabolism could play a substantial role. Finally, also the iron content of adipocytes should be investigated. Indeed, Zhang et al. [37] showed that lowering iron in adipocytes protects mice from high-fat-diet-induced metabolic dysfunction, suggesting that iron content can be used as a sensor to activate an adipose–gut cross talk to regulate lipid absorption.

This study has several notable strengths. The use of real-world clinical data enhances the external validity and applicability of our findings. Additionally, the large sample size provides sufficient statistical power, increasing the reliability and robustness of the results. The comprehensive profiling of participants—including anthropometric, biochemical, and imaging parameters—represents a reproducible assessment of the associations between serum iron levels and cardiometabolic risk factors. Importantly, stratified analyses revealed significant sex-specific differences, particularly regarding the correlation between circulating iron and obesity-related measures in women. These findings further support the emerging concept linking iron metabolism and ferroptosis-mediated inflammation as pivotal contributors to metabolic dysfunction and its clinical manifestations.

However, several limitations should be acknowledged. The degree of systemic inflammation may have influenced iron status; unfortunately, key inflammatory biomarkers involved in the iron homeostasis-inflammation axis—such as interleukin-6, hepcidin, and soluble transferrin receptor—were not available for analysis. Moreover, ethnic differences may limit the worldwide generalizability of our results, especially considering the influence of environmental factors such as dietary habits and pollution, which are known to contribute to both metabolic and cardiovascular diseases. Finally, the lack of information about menstrual status does not allow to precisely depict the iron status of women in our cohort.

## Conclusions

Our study underscores the potential utility of serum iron as a biomarker for the evaluation of patients with MetS and its related clinical conditions. Within our cohort, reduced serum iron levels were consistently associated with T2D and arterial hypertension, two central components of MetS. Notably, these associations extended to glucose, HbA1c, and lipid panel, suggesting that alterations in iron status may reflect the convergence of multiple metabolic pathways. Surprisingly, while no significant correlations were detected between iron and WC and BMI in the overall cohort, such correlations were found to be significant only in women, supporting the hypothesis that iron metabolism and related pathophysiological mechanisms differ between men and women. These insights may have important implications for risk stratification and personalized management strategies, particularly in women presenting with early metabolic disturbances.

Clinically, our findings advocate for a more nuanced interpretation of serum iron levels in patients exhibiting features of MetS. Assessing iron status could be particularly valuable for risk stratification in high-risk individuals, such as women with obesity. Moreover, although it may be premature to recommend iron supplementation based only on our findings, our finding encourages further research into iron-related pathways as putative novel diagnostic and therapeutic hits in metabolic diseases.

## Supplementary Information

Below is the link to the electronic supplementary material.

Supplementary file1 (DOCX 74 KB)

Supplementary file2 (DOCX 721 KB)

Supplementary file3 (DOCX 15 KB)

## Data Availability

Data presented in this study are available on request from the corresponding author.

## References

[1] Mancardi D, Mezzanotte M, Arrigo E, Barinotti A, Roetto A (2021) Iron overload, oxidative stress, and Ferroptosis in the failing heart and liver. Antioxidants (Basel) 10(12):18647 10.3390/antiox10121864 PMC8698778 34942967

[2] Marti-Aguado D, Ten-Esteve A, Baracaldo-Silva CM, Crespo A, Coello E, Merino-Murgui V et al (2023) Pancreatic steatosis and iron overload increases cardiovascular risk in non-alcoholic fatty liver disease. Front Endocrinol (Lausanne) 14:1213441 37600695 10.3389/fendo.2023.1213441 PMC10436077

[3] Li D, Jiang C, Mei G, Zhao Y, Chen L, Liu J et al (2020) Quercetin alleviates Ferroptosis of pancreatic β cells in type 2 diabetes. Nutrients 12(10):2954 32992479 10.3390/nu12102954 PMC7600916

[4] Capelletti MM, Manceau H, Puy H, Peoc’h K (2020) Ferroptosis in liver diseases: an overview. Int J Mol Sci 21(14):4908 32664576 10.3390/ijms21144908 PMC7404091

[5] National Cholesterol Education Program (NCEP) (2002) Expert panel on detection, evaluation, and treatment of high blood cholesterol in adults (Adult Treatment Panel III). Third report of the national cholesterol education program (NCEP) expert panel on detection, evaluation, and treatment of high blood cholesterol in adults (Adult Treatment Panel III) final report. Circulation 106(25):3143–3421 12485966

[6] Samson SL, Garber AJ (2014) Metabolic syndrome. Endocrinol Metab Clin North Am 43(1):1–23 24582089 10.1016/j.ecl.2013.09.009

[7] Wang TY, George J, Zheng MH (2021) Metabolic (dysfunction) associated fatty liver disease: more evidence and a bright future. Hepatobiliary Surg Nutr 10(6):849–852 35004952 10.21037/hbsn-21-352 PMC8683909

[8] Wu S, Xu W, Guan C, Lv M, Jiang S, Jinhua Z (2023) Global burden of cardiovascular disease attributable to metabolic risk factors, 1990–2019: an analysis of observational data from a 2019 Global Burden of Disease study. BMJ Open 13(5):e069397 37173115 10.1136/bmjopen-2022-069397 PMC10186407

[9] Gosadi IM (2016) Assessment of the environmental and genetic factors influencing prevalence of metabolic syndrome in Saudi Arabia. Saudi Med J 37(1):12–20 26739969 10.15537/smj.2016.1.12675 PMC4724673

[10] Suárez-Ortegón MF, Ensaldo-Carrasco E, Shi T, McLachlan S, Fernández-Real JM, Wild SH (2018) Ferritin, metabolic syndrome and its components: a systematic review and meta-analysis. Atherosclerosis 275:97–106 29886355 10.1016/j.atherosclerosis.2018.05.043

[11] Kaiafa G, Kanellos I, Savopoulos C, Kakaletsis N, Giannakoulas G, Hatzitolios AI (2015) Is anemia a new cardiovascular risk factor? Int J Cardiol 186:117–124 25814357 10.1016/j.ijcard.2015.03.159

[12] Do HJ, Lee YS, Ha MJ, Cho Y, Yi H, Hwang YJ et al (2016) Beneficial effects of voglibose administration on body weight and lipid metabolism via gastrointestinal bile acid modification. Endocr J 63(8):691–702 27349182 10.1507/endocrj.EJ15-0747

[13] Anker SD, Comin Colet J, Filippatos G, Willenheimer R, Dickstein K, Drexler H et al (2009) Ferric carboxymaltose in patients with heart failure and iron deficiency. N Engl J Med 361(25):2436–2448 19920054 10.1056/NEJMoa0908355

[14] Rajendran P, Rengarajan T, Thangavel J, Nishigaki Y, Sakthisekaran D, Sethi G et al (2013) The vascular endothelium and human diseases. Int J Biol Sci 9(10):1057–1069 24250251 10.7150/ijbs.7502 PMC3831119

[15] Gill D, Del Greco MF, Walker AP, Srai SKS, Laffan MA, Minelli C (2017) The effect of iron status on risk of coronary artery disease: a mendelian randomization study-brief report. Arterioscler Thromb Vasc Biol 37(9):1788–1792 28684612 10.1161/ATVBAHA.117.309757

[16] Birben E, Sahiner UM, Sackesen C, Erzurum S, Kalayci O (2012) Oxidative stress and antioxidant defense. World Allergy Organ J 5(1):9–19 23268465 10.1097/WOX.0b013e3182439613 PMC3488923

[17] Coban E, Ozdogan M, Timuragaoglu A (2004) Effect of iron deficiency anemia on the levels of hemoglobin A1c in nondiabetic patients. Acta Haematol 112(3):126–128 15345893 10.1159/000079722

[18] Elsheikh E, Aljohani SS, Alshaikhmubarak MM, Alhawl MA, Alsubaie AW, Alsultan N et al (2023) Implications of iron deficiency anaemia on glycemic dynamics in diabetes mellitus: a critical risk factor in cardiovascular disease. Cureus 15(11):e49414 38149144 10.7759/cureus.49414 PMC10750114

[19] Effect of iron deficiency anemia on blood glucose and insulin resistance in women with type II diabetes: a single-group, Clinical Interventional Study - PubMed [Internet]. [citato 9 giugno 2025]. Disponibile su: https://pubmed.ncbi.nlm.nih.gov/38561621/10.2174/0115748871297808240308102327 38561621

[20] Pradeepa R, Shreya L, Anjana RM, Jebarani S, Kamal Raj N, Kumar MS et al (2022) Frequency of iron deficiency anemia in type 2 diabetes - Insights from tertiary diabetes care centres across India. Diabetes Metab Syndr 16(11):102632 36343394 10.1016/j.dsx.2022.102632

[21] Levi M, Simonetti M, Marconi E, Brignoli O, Cancian M, Masotti A et al (2019) Gender differences in determinants of iron-deficiency anemia: a population-based study conducted in four European countries. Ann Hematol 98(7):1573–1582 31073646 10.1007/s00277-019-03707-w

[22] Chen Z, Cao B, Liu L, Tang X, Xu H (2024) Association between obesity and anemia in an nationally representative sample of United States adults: a cross-sectional study. Front Nutr 11:1304127 38544758 10.3389/fnut.2024.1304127 PMC10965637

[23] Aguree S, Owora A, Hawkins M, Reddy MB (2023) Iron deficiency and iron deficiency anemia in women with and without obesity: NHANES 2001–2006. Nutrients 15(10):2272 37242155 10.3390/nu15102272 PMC10223101

[24] Crudele L, Piccinin E, Moschetta A (2021) Visceral adiposity and cancer: role in pathogenesis and prognosis. Nutrients 13(6):2101 34205356 10.3390/nu13062101 PMC8234141

[25] Moreno-Navarrete JM, Ortega F, Moreno M, Ricart W, Fernández-Real JM (2014) Fine-tuned iron availability is essential to achieve optimal adipocyte differentiation and mitochondrial biogenesis. Diabetologia 57(9):1957–1967 24973963 10.1007/s00125-014-3298-5

[26] González-Domínguez Á, Visiedo-García FM, Domínguez-Riscart J, González-Domínguez R, Mateos RM, Lechuga-Sancho AM (2020) Iron metabolism in obesity and metabolic syndrome. Int J Mol Sci 21(15):5529 32752277 10.3390/ijms21155529 PMC7432525

[27] Tussing-Humphreys LM, Nemeth E, Fantuzzi G, Freels S, Guzman G, Holterman AXL et al (2010) Elevated systemic hepcidin and iron depletion in obese premenopausal females. Obesity (Silver Spring) 18(7):1449–1456 19816411 10.1038/oby.2009.319

[28] Kong WN, Niu QM, Ge L, Zhang N, Yan SF, Chen WB et al (2014) Sex differences in iron status and hepcidin expression in rats. Biol Trace Elem Res 160(2):258–267 24962641 10.1007/s12011-014-0051-3

[29] Bekri S, Gual P, Anty R, Luciani N, Dahman M, Ramesh B et al (2006) Increased adipose tissue expression of hepcidin in severe obesity is independent from diabetes and NASH. Gastroenterology 131(3):788–796 16952548 10.1053/j.gastro.2006.07.007

[30] Ganz T, Nemeth E (2015) Iron homeostasis in host defence and inflammation. Nat Rev Immunol 15(8):500–510 26160612 10.1038/nri3863 PMC4801113

[31] Savarese G, von Haehling S, Butler J, Cleland JGF, Ponikowski P, Anker SD (2022) Iron deficiency and cardiovascular disease. Eur Heart J 44(1):14–27 10.1093/eurheartj/ehac569 PMC9805408 36282723

[32] Fernández-Real JM, López-Bermejo A, Ricart W (2002) Cross-talk between iron metabolism and diabetes. Diabetes 51(8):2348–2354 12145144 10.2337/diabetes.51.8.2348

[33] Leiva E, Mujica V, Sepúlveda P, Guzmán L, Núñez S, Orrego R et al (2013) High levels of iron status and oxidative stress in patients with metabolic syndrome. Biol Trace Elem Res 151(1):1–8 23079936 10.1007/s12011-012-9525-3

[34] Park SK, Ryoo JH, Kim MG, Shin JY (2012) Association of serum ferritin and the development of metabolic syndrome in middle-aged Korean men: a 5-year follow-up study. Diabetes Care 35(12):2521–2526 22933431 10.2337/dc12-0543 PMC3507565

[35] Yu L, Que T, Zhou Y, Liu Z (2024) Dose-response relationship of serum ferritin and dietary iron intake with metabolic syndrome and non-alcoholic fatty liver disease incidence: a systematic review and meta-analysis. Front Nutr 11:1437681 39410926 10.3389/fnut.2024.1437681 PMC11476413

[36] Lu CW, Lee YC, Kuo CS, Chiang CH, Chang HH, Huang KC (2021) Association of serum levels of zinc, copper, and iron with risk of metabolic syndrome. Nutrients 13(2):548 33562398 10.3390/nu13020548 PMC7914992

[37] Zhang Z, Funcke JB, Zi Z, Zhao S, Straub LG, Zhu Y et al (2021) Adipocyte iron levels impinge on a fat-gut crosstalk to regulate intestinal lipid absorption and mediate protection from obesity. Cell Metab 33(8):1624-1639.e9 34174197 10.1016/j.cmet.2021.06.001 PMC8338877

