# Supplementary figures and images for: Reduced serum iron levels are associated with metabolic dysfunction and sex-specific characteristics
Source: Intern Emerg Med. 2025 Nov 6;21(1):129–39. doi: 10.1007/s11739-025-04169-x (PMC12948878; doi:10.1007/s11739-025-04169-x)

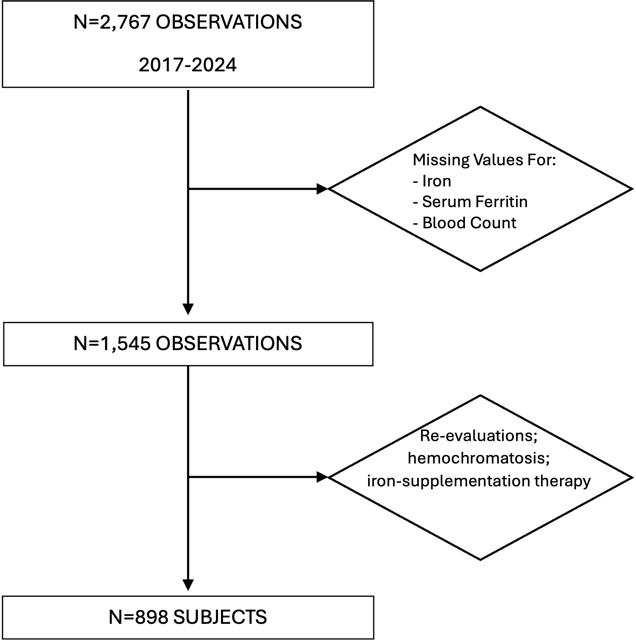


**Supplementary Figure 1. Study design.**

Supplement: Supplementary file 1 — Supplementary file1 (DOCX 74 KB) [file 11739_2025_4169_MOESM1_ESM.docx]
