# Supplementary material for: Reduced serum iron levels are associated with metabolic dysfunction and sex-specific characteristics
Source: Intern Emerg Med. 2025 Nov 6;21(1):129–39. doi: 10.1007/s11739-025-04169-x (PMC12948878; doi:10.1007/s11739-025-04169-x)
Supplement: Supplementary file 2 — Supplementary file2 (DOCX 721 KB) [file 11739_2025_4169_MOESM2_ESM.docx]

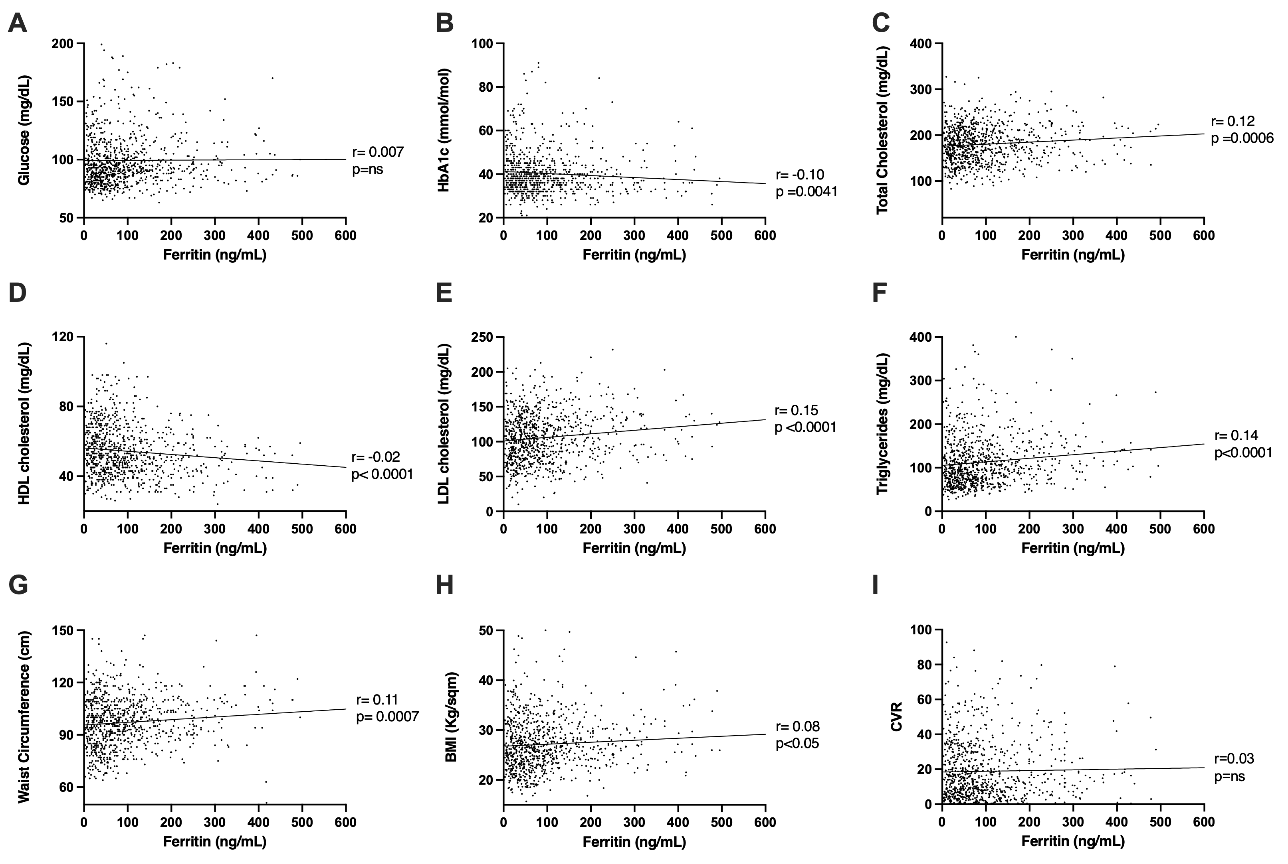


**Supplementary Figure 2. Serum ferritin levels correlations with bio-humoral and clinical parameters in the study population.**

Pearson’s correlations (r) and corresponding p-values (p). Abbreviations: CVR, cardiovascular risk assessed by Framingham Risk Score.
