# Supplementary material for: Reduced serum iron levels are associated with metabolic dysfunction and sex-specific characteristics
Source: Intern Emerg Med. 2025 Nov 6;21(1):129–39. doi: 10.1007/s11739-025-04169-x (PMC12948878; doi:10.1007/s11739-025-04169-x)
Supplement: Supplementary file 3 — Supplementary file3 (DOCX 15 KB) [file 11739_2025_4169_MOESM3_ESM.docx]

**Supplementary Table 1.**

| **Variable** | **Correlation Coefficient** | **p-value** |
| --- | --- | --- |
| FPG | -0.19 | <0.0001 |
| HbA1c | -0.27 | <0.0001 |
| Total cholesterol | 0.16 | 0.0083 |
| HDL-cholesterol | 0.11 | 0.0392 |
| LDL-cholesterol | 0.22 | <0.0001 |
| Triglycerides | 0.04 | 0.5932 |
| Waist circumference | -0.17 | 0.0071 |
| BMI | -0.09 | 0.0873 |

Pearson’s partial correlation coefficients between iron levels and various clinical parameters, adjusted for serum ferritin.
